# Papillary craniopharyngioma management in the era of BRAF and MEK inhibition

**DOI:** 10.1007/s11060-025-04969-3

**Published:** 2025-02-20

**Authors:** Mark Damante, Santino Cua, Daniel Kreatsoulas, Pierre Giglio, Luma Ghalib, Chandrima Biswas, Kyle C. Wu, Daniel M. Prevedello

**Affiliations:** 1 Department of Neurological Surgery, The Ohio State University Wexner Medical Center, 410 W 10th Ave N1019 Doan Hall, Columbus, OH 43210 USA; 2 Division of Neuro Oncology, The Ohio State University Wexner Medical Center, Columbus, OH USA; 3 Division of Endocrinology, The Ohio State University Wexner Medical Center, Columbus, OH USA

**Keywords:** Papillary craniopharyngioma, BRAF V600E mutation, Targeted therapy, Radiation

## Abstract

**Purpose:**

Papillary craniopharyngioma is a rare entity, demonstrating BRAF-V600E mutations in approximately 95% of patients. Recently, a phase 2 trial of patients treated with surgery and BRAF/MEKi demonstrated 91% reduction in residual tumor volume. This study allowed for additional treatments at the discretion of the treatment team without reporting subsequent rates of endocrinopathy or visual decline. We aimed to evaluate the possibility of employing BRAF/MEKi without the need for adjuvant radiotherapy therapies.

**Methods:**

A retrospective report of two patients treated with resection and BRAF/MEKi without additional treatment were analyzed. Patient demographics, treatment characteristics, pre- and post-treatment radiographic volumes, adverse events, and endocrinologic and visual outcomes, were recorded and analyzed.

**Results:**

Two patients underwent subtotal resection followed by BRAF/MEKi without adjuvant treatment. Mean length of BRAF therapy was 21.4 months and MEKi therapy was 12.94 months. Mean preoperative nodule volume was 0.33 cm [3] and 2.29 cm [3] and cystic volume was 5.04 cm [3] and 6.18 cm [3] in case 1 and case 2, respectively. Neither patient received radiation. Grade 3 cardiotoxicity developed in case 1 after 6.5 months, with function recovering completely following discontinuation of MEKi. BRAF therapy was discontinued electively after 23.5 months. The second patient remains on dual inhibition therapy without toxicity. For these cases, post-treatment nodule volumes are 0.07 cm [3] (98.4% reduction) and 0.04 cm [3] (99.2% reduction), respectively, and cystic volume 0.0 cm [3] in both patients. Progression free survival is 100% with a mean follow up of 36-months.

**Conclusions:**

Utilizing surgery and BRAF/MEKi without adjuvant radiation, we demonstrate excellent disease control with reversible toxicity. Avoiding additional treatments may spare vital functions and unnecessary procedures.

## Introduction

Craniopharyngioma makes up approximately 2–5% of intracranial tumors. In 1984, craniopharyngioma was separated into two distinct histopathologic subtypes: adamantinomatous and papillary [1, 2]. This distinction in craniopharyngioma is also observed radiographically, with lack of calcification suggesting papillary craniopharyngioma. In 2015, Brastianos et al. identified via whole exome sequencing analysis, that, as many as, 95% of papillary craniopharyngioma contain the BRAF V600E mutation [3]. This unique molecular signature led to the reclassification of adamantinomatous (CTNNB1) and papillary (BRAF) craniopharyngiomas as different pathologic entities by the Word Health Organization (WHO) in 2021 [4]. 

Prior to this genomic distinction between craniopharyngiomas, standard practice consisted of maximally safe resection followed by radiotherapy for control of residual disease, and this remains true in adamantinomatous craniopharyngioma [5, 6]. However, despite a less aggressive surgical approach to avoid pituitary damage, adjuvant radiotherapy, in the form of either conventional radiotherapy or stereotactic radiosurgery, is associated with up to 20% risk of pituitary disfunction [5]. Improvement in radiotherapy techniques have allowed for highly conformal delivery, thereby decreasing exposure of the pituitary, hypothalamus and optic nerve to higher marginal doses of radiation [7, 8]. Proton beam therapy (PBT) has become increasingly utilized for treatment of craniopharyngioma for this same reason due to the theoretical benefits of the brag peak and advances in scanning pencil beam delivery compared to the traditional passive scatter proton beam therapy [9–12]. With these radiotherapy techniques, overall and progression free survival following subtotal resection does not significantly differ from upfront gross total resection. However, progressive endocrinologic deficits, hypothalamic dysfunction, cognitive decline, cerebral vasculopathy and secondary malignancy persist with significant impact on quality-of-life outcomes [13]. Systemic therapy is not yet standard of care.

The consistency of the BRAF V600E mutation makes it an ideal target for molecular targeted therapies, known collectively as BRAF inhibitors, as well as MEK inhibitors, a downstream target of the *ras* oncogene pathway [14]. These therapies have been successfully implemented in melanoma and melanoma brain metastases with acceptable side effect profiles, but application to craniopharyngioma had been limited to off-label use at treating oncologist’s discretion [15]. Brastianos et al. published the results of a phase 2 nonrandomized trial containing 16 patients with papillary craniopharyngioma treated with surgical resection and adjuvant BRAF/MEKi.^16^ Adjuvant radiotherapy was allowed at the discretion of the treating physician and details were not reported [16]. An extreme example of the potential success of BRAF ± MEK inhibition in papillary craniopharyngioma is demonstrated in a case report of a patient with radiographic evidence of craniopharyngioma, but refusal of surgical resection or biopsy for tissue diagnosis [17]. This patient was treated with upfront BRAF inhibition with subsequent rapid radiographic response in just 19 days and complete radiographic response during the 6.5 month follow up period [17]. 

While empiric treatment using a BRAF/MEKi is risky to subject a patient to, given the potential for incorrect diagnosis and/or adverse medication effects, their success in the current literature is undeniable and further investigation is critical to determining the optimal implantation strategy. The present study demonstrates the oncologic outcomes of two patients consecutively treated with subtotal surgical resection followed by at least six months of BRAF/MEKi therapy, while avoiding radiation therapy and explores additional potential strategies to consider in the BRAF/MEK inhibitor era.

## Methods

Two consecutive patients with papillary craniopharyngioma treated with subtotal resection and adjuvant BRAF/MEKi, without radiosurgery, were identified and analyzed in a multi-institutional retrospective case series. Patient demographics were recorded. Preoperative and postoperative imaging characteristics were obtained, including pre- and post-operative volumetric tumor analysis, location of tumor and location of residual tumor, type of residual (cystic or nodular), and stability/resolution of tumor residual. Volumetric analysis was performed M.A.D and K.C.W using BrainLab software (Brainlab Elements version 3.1.0; Brainlab AG, Munich, Germany) and analyzed the volume of the contrast enhancing nodular component and/or the volume of the cystic component. Surgical intervention data was recorded including type of approach (endonasal or transcranial) and perioperative complications. Pre- and post-operative endocrinopathy, visual disturbance and other neurologic deficits were also compared. Systemic treatment information including BRAF/MEKi agent(s) used, duration of treatment, total cycles, grade 3 and 4 adverse events as defined by CTCAE, and alterations in systemic treatment dosage and medication were analyzed. Data analysis was performed with SPSS (IBM Corp. Released 2023. IBM SPSS Statistics for Macintosh, Version 29.0.2.0 Armonk, NY: IBM Corp).

## Results

### Case 1

**Fig. 1:**
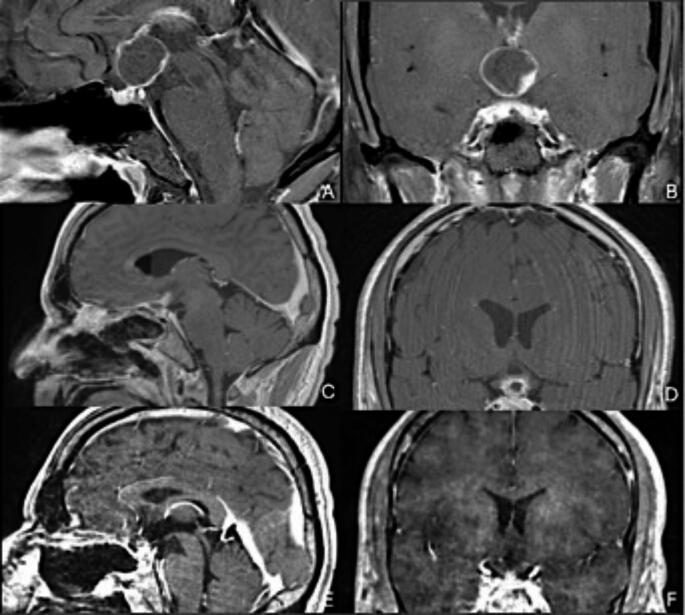
Panel A-B show preoperative MRI with a complex solid-cystic suprasellar lesion without calcification. Panel C-D show the immediate postoperative MRI demonstrating decompression of the cystic component of the tumor with residual enhancing tumor along the optic chiasm following endoscopic endonasal surgery for decompression and diagnosis. Panel E-F show an MRI at most recent follow up after treatment with combined BRAF/MEK inhibitor therapy with minimal residual enhancement, which could be gliotic tissue versus miniscule residual nodular component of the tumor

A 40-year-old male patient presented to clinic with six months of blurry vision. Formal visual field testing delineated bitemporal hemianopsia. He then underwent a magnetic resonance imaging (MRI) of the MRI brain with and without contrast demonstrating a non-calcified, suprasellar mass with both solid and cystic components (Fig. 1**)**. The cystic component volume was 5.04 cm [3] and nodular volume was 0.33 cm [3]. The patient underwent endocrinologic workup consistent with a mild diabetes insipidus that was managed medically. Surgical resection was offered, and the patient underwent an endoscopic endonasal transsellar and trans-tuberculum approach for subtotal resection of the lesion where it was adherent to visual apparatus and pituitary stalk. Post-operative MRI showed residual tumor along the optic chiasm and infundibulum, as expected. The postoperative enhancing volume was 0.72 cm [3], an 85.7% extent of resection. Histopathologic and genomic analyses were consistent with papillary craniopharyngioma harboring the BRAF V600E mutation. The patient’s bitemporal hemianopsia subjectively improved immediately postop and at one month follow up was entirely recovered. He was started on low dose DDAVP and levothyroxine for mild DI and hypothyroidism.

After discussion of the patient’s case at multidisciplinary tumor board, the decision was made to start the patient on dual BRAF/MEK inhibitor therapy (dabrafenib/trametinib). To date, the patient has been on therapy for a total of 19 months without any systemic toxicity as defined by CTCAE criteria. Serial MRIs have been performed with complete resolution of the cystic component and a small 0.07 cm [3] residual enhancement at the optic chiasm, equating to an additional 98.4% reduction from the residual postoperative tumor volume. The patient has not been treated with radiotherapy due to his durable response to BRAF/MEK inhibition thus far.

### Case 2

**Fig. 2:**
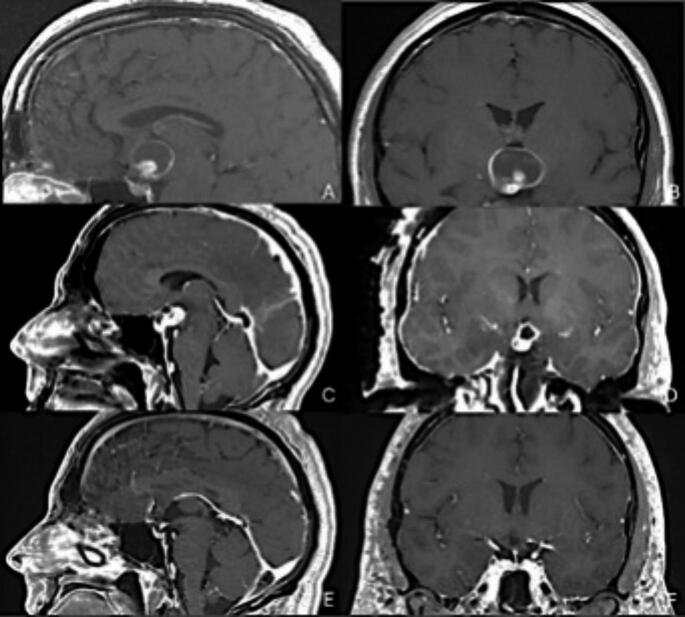
Panel A-B show preoperative MRI with a complex solid-cystic intraventricular lesion without calcification. Panel C-D show the immediate postoperative MRI demonstrating decompression of the cystic component of the tumor with residual enhancing tumor along the optic chiasm and hypothalamus following right sided cranioorbital craniotomy subfrontal approach for decompression and diagnosis. Panel E-F show an MRI at most recent follow up after treatment with combined BRAF/MEK inhibitor therapy, and ultimately discontinuation of both, with minimal residual enhancement

A 46-year-old male presented to clinic with six months of fatigue, 20–30-pound weight gain and polydipsia and polyuria with new nocturia. Pituitary labs demonstrated complete panhypopituitarism requiring desmopressin, levothyroxine and cortisol replacement. Formal visual field testing did not reveal any visual deficits. An MRI brain was obtained and demonstrated a retrochiasmatic, non-calcified mass with both solid and cystic components (Fig. 2**)**. The preoperative cystic component volume was 6.18 cm [3] and the nodular component was 2.29 cm [3]. The patient underwent a craniotomy for a subfrontal, trans-lamina terminals approach to a retrochiasmatic lesion. A subtotal resection was performed due to adherence of tumor to the hypothalamus. Postoperative MRI brain confirmed the hypothalamic remnant to be the only residual. Postoperative enhancing volume was 1.23 cm [3], an 80.1% extent of resection. The patient’s recovery was uneventful. Histopathologic and genomic analysis of the lesion was consistent with papillary craniopharyngioma with the BRAF V600E mutation. The patient’s case was discussed at the multidisciplinary tumor board, and it was decided to initiate dual BRAF/MEK inhibitor therapy (dabrafenib/trametinib). The patient completed 6.5 months (seven cycles), at which point, he began developing symptoms of cardiomyopathy, which was confirmed by a reduction in ejection fraction to 35% on transthoracic echocardiogram. The decision was made to discontinue the trametinib, which resulted in complete recovery of cardiac function. He continued dabrafenib therapy alone for a total of 23.5 months, at which point the patient chose to discontinue therapy. At this point, he had stable MRI scans with only a 0.04 cm [3] residual at the hypothalamus, equating to an additional 99.2% reduction from the residual postoperative tumor volume. The patient has now been off systemic therapy for 26.4 months without recurrence on MRI. Total follow up has been 51 months. The patient has not been treated with radiotherapy due to his durable response to BRAF/MEK inhibition, even after discontinuation.

## Discussion

Genomic sequencing has led to the discovery of targetable tumor mutations resulting in significant improvements in efficacy of targeted therapies and altering oncologic treatment paradigms. The BRAF V600E mutation has been identified in multiple tumors, including melanoma, pleomorphic xanthoastrocytoma (PXA), ganglioglioma and papillary craniopharyngioma [3, 15, 18, 19]. Melanoma is, perhaps, the most recognizable success of BRAF inhibition, with randomized clinical trials comparing BRAF and MEK inhibition to previous generation cytotoxic and small molecule inhibitor agents [20]. In the past decade, the strong association between the BRAF V600E mutation and papillary craniopharyngioma diagnosis has prompted discussion regarding utilization of BRAF and MEK inhibitors as treatment.

Recently, Brastianos et al. completed the first non-randomized clinical trial studying safety and efficacy of combined BRAF and MEK inhibition in histopathological and genome sequencing proven V600E mutated papillary craniopharyngioma. Their study is the first of its kind and efforts in patient accrual should be commended given the rare nature of the disease. The study shows excellent tumoral response to a 28-day course of adjuvant combined BRAF and MEK inhibitor therapy following surgical resection. Due to toxicity limitations, three patients had to discontinue therapy prematurely. Post-therapy volumetric analysis demonstrated response in both nodular enhancing and cystic portions of residual tumor with 87% progression free survival at 12-months and 58% progression free survival at 24 months. It is unclear if there were any post-BRAF/MEK inhibition therapy endocrinopathies, though this is not a previously described adverse effect of the medications. The study provides preliminary prospective data in favor of BRAF and MEK inhibition for the treatment of papillary craniopharyngioma. However, multiple questions surrounding BRAF and MEK inhibitor therapy for this disease remain, particularly surrounding necessity of adjuvant radiosurgery, duration of treatment in patients without adverse events, and the role of neurosurgical management.

Given that craniopharyngioma originates from the pituitary infundibulum, 40–87% of patients present with at least one endocrine deficiency [21, 22]. However, in those where at least some degree of gland function is retained, surgical intervention is aimed at maximal safe resection while preserving the infundibulum and hypothalamus to avoid iatrogenic panhypopituitarism. Postoperative radiation is often delivered to maximize control of the residual component of tumor, however, up to 94% of patients demonstrate radiation induced new or progressive endocrinopathy over the following five years [10, 21]. Avoidance of the need for adjuvant radiation therapy by controlling disease with adjuvant BRAF and MEK inhibition may deliver better endocrinologic outcomes. While the patient in case 2 had poor pituitary function at presentation, we were able to easily preserve the baseline function for the patient in case 1 by pursuing a subtotal resection followed by BRAF and MEK inhibition without adjuvant radiation. Evidence of tumor progression at some point in the disease process may necessitate salvage radiation therapy, but one could also consider a longer treatment course than the 28-day course described in the trial, or retreatment with surgery or BRAF/MEKi which may result in continued disease control while sparing side effects of radiation.

The presented two patients received 19.3 and 23.7 months of adjuvant BRAF/MEK inhibitor therapy with no evidence of recurrence at 22.3 and 51.2 months follow up, respectively. Understandably, not all patients will tolerate a prolonged course of therapy, as grade 3 and 4 CTCAE adverse events occur in approximately 46% of patients, though are typically transient with discontinuation or reduction of therapy [23]. Index case 2 developed cardiotoxicity with a significant reduction in ejection fraction that resolved after discontinuation of the MEK inhibitor. He continued on BRAF inhibition only for an additional 17 months before electing to cease the medication. While longer therapy times may potentially improve control, continued tumor control on monotherapy proposes an alternative treatment paradigm. This also brings to attention the necessity of upfront dual versus monotherapy with BRAF and/or MEK inhibition. Neither patient experienced a decline in endocrine function following initiation of treatment.

It is well described that, unlike adamantinomatous craniopharyngioma, papillary craniopharyngioma will not demonstrate calcification on radiographic study. Often, the diagnosis of papillary craniopharyngioma is correctly presumed preoperatively, with up to 90% accuracy in one study [24]. A case report by Lin et al. describes a patient with a presumed papillary craniopharyngioma diagnosis in a patient that ultimately refused surgery and was subsequently treated empirically with combined BRAF and MEK inhibition, demonstrating complete response, but with only two month follow up at time of publication [17]. While empiric treatment in this unique case was effective, tissue diagnosis is still recommended for formal diagnosis and molecular and genetic analysis, as approximately 5% of papillary craniopharyngiomas have not demonstrated the BRAF V600E mutation, as described in the original genomic analysis [3]. 

Based on the safety and efficacy of BRAF/MEK inhibitor therapy, exploration of new treatment paradigms is critical to maximizing patient outcomes. The excellent control rates with BRAF/MEK inhibitor therapy demonstrated in these two cases and in the Brastianos clinical trial suggest consideration of a more conservative treatment course. In the two presented cases we determined preoperatively that surgical goal was diagnosis for BRAF V600E mutation and only debulking of tumor that could be readily resected without need for significant dissection off critical neurovascular structures. An argument could be made to suggest maximal safe resection in all cases, as smaller postoperative residual may increase effectiveness of targeted therapy or reduce doses required to see an effect, though a substantial response was noted in both presented cases with conservative resection. Alternatively, one case report of a single patient’s experience describes empiric treatment with BRAF/MEK inhibition alone of a presumed papillary craniopharyngioma with excellent radiographic and neuroendocrinologic outcomes [17]. However, while empiric treatment is enticing to avoid surgical intervention entirely, it is cautioned, as approximately 5% of histopathologic- and radiographic-appearing craniopharyngioma do not harbor the BRAF V600E mutation.

In both presented cases, subtotal resection for decompression of optic apparatus was performed followed by a prolonged course of BRAF/MEK inhibition without need for adjuvant radiotherapy. This is in contrast to a patient previously treated by our group prior to more routine BRAF/MEK inhibitor utilization, who presented with normal pituitary function. While surgery was technically uncomplicated and gross total resection was achieved without intraoperative concern for compromise of the pituitary gland, infundibulum or hypothalamus, the patient became profoundly deficient in all pituitary hormones. This highlights an important point of discussion where despite preoperative characteristics favoring total resection with minimal neuroendocrinologic morbidity (such as Puget grade 0 lesions) [25], the risk is not zero. It is possible that a greater response to BRAF/MEKi may be observed following a greater degree of surgical cytoreduction but requires risk-benefit analysis. A more conservative approach hinging on response to BRAF/MEK inhibitor therapy may have spared her the postoperative panhypopituitarism. We recently treated a fourth case in which there was a high preoperative probability of papillary craniopharyngioma. This diagnosis was confirmed on molecular sequencing following an endonasal approach for cyst decompression and subtotal resection of the lesion. The patient had no preoperative or postoperative endocrinopathy. Given the elderly age of the patient, the decision has been made to defer all additional therapy until there is evidence of radiographic progression or new neuroendocrinologic deficits.

The present series does not aim to suggest a stringent treatment paradigm to be followed by all who treat papillary craniopharyngioma. There is much to be learned about the papillary craniopharyngioma pathology and its natural history. Our group has had success in managing such patients in the BRAF/MEK inhibitor era compared to patients treated prior to. The observed success in the few patients we have treated has made us less aggressive in initial surgical management and more reluctant to pursue adjuvant radiation in suspected papillary craniopharyngioma. However, it should be noted that our team is located at a high-volume skull base practice, in which the neuro-oncology, radiation oncology, endocrinology and neurosurgeons are comfortable with pursuing less aggressive management upfront, as there is ample experience with potential need for salvage resection and/or radiosurgery in cases of progression despite targeted therapy. While generalizable conclusion cannot be drawn from the index cases, as BRAF/MEK inhibitors become more frequently used and studied, evidence will likely continue to favor trials of adjuvant therapy while we continue to evaluate the most effective and safest implementation.

## Conclusion

The discovery of the BRAF V600E mutation in majority of papillary craniopharyngioma tumors offers numerous potential adjustments to current standard treatment algorithms, which often include surgical resection and adjuvant radiotherapy. We describe our current considerations for management of tumors with high preoperative concern for the papillary craniopharyngioma diagnosis. Tissue confirmation followed by initiation of dual BRAF/MEK inhibition for at least 6-months, if tolerated, while avoiding adjuvant radiotherapy in absence of radiographic progression may lead to both better oncologic, visual, and neuroendocrinologic outcomes. There are multiple subtleties to these evolving treatment paradigms that have yet to be answered, including utility of dual versus monotherapy and exact duration of treatment necessary to obtain a durable response.

## Data Availability

No datasets were generated or analysed during the current study.
